# Novel clostridial lineages recovered from metagenomes of a hot oil reservoir

**DOI:** 10.1038/s41598-020-64904-6

**Published:** 2020-05-15

**Authors:** Glenn D. Christman, Rosa I. León-Zayas, Rui Zhao, Zarath M. Summers, Jennifer F. Biddle

**Affiliations:** 1 School of Marine Science and Policy, University of Delaware, Lewes, DE USA; 2 Department of Biological Sciences, Willamette University, Salem, OR USA; 3 ExxonMobil Research and Engineering, Annandale, NJ USA

**Keywords:** Biogeochemistry, Environmental microbiology

## Abstract

Oil reservoirs have been shown to house numerous microbial lineages that differ based on the *in-situ* pH, salinity and temperature of the subsurface environment. Lineages of Firmicutes, including Clostridiales, have been frequently detected in oil reservoirs, but are typically not considered impactful or relevant due to their spore-forming nature. Here we show, using metagenomics, a high temperature oil reservoir of marine salinity contains a microbial population that is predominantly from within the Order Clostridiales. These organisms form an oil-reservoir specific clade based on the phylogenies of both 16S rRNA genes and ribosomal proteins, which we propose to name ^U^*Petromonas tenebris*, meaning they are single-celled organisms from dark rocks. Metagenome-assembled genomes (MAGs) of these *Petromonas* sp. were obtained and used to determine that these populations, while capable of spore-formation, were also likely replicating *in situ* in the reservoir. We compared these MAGs to closely related genomes and show that these subsurface Clostridiales differ, from the surface derived genomes, showing signatures of the ability to degrade plant-related compounds, whereas subsurface genomes only show the ability to process simple sugars. The estimation of *in-situ* replication from genomic data suggest that ^U^*Petromonas tenebris* lineages are functional *in-situ* and may be specifically adapted to inhabit oil reservoirs.

## Introduction

Oil reservoirs are unique subsurface environments that can be hostile to life due to high temperatures, pressures, and salinity (reviewed in^1^). It is thought that temperatures in source rocks or reservoirs greater than 80 °C act to deter hydrocarbon degradation and sterilize microbial life^2,3^. As sterile crude oil and gases migrate from the hot source rocks and cool they accumulate and may be populated by existing subsurface communities in rock or flowing subterranean waters. Determining the indigenous microbial community structures in these reservoirs can be difficult because of the presence of pipeline and infrastructure contaminants, and the indigenous communities themselves can be altered in terms of member representation and activity by the injection of seawater or gases and chemical additives used for secondary recovery^4^.

Microbes can cause souring or consume significant quantities of lower molecular weight hydrocarbons, degrading the quality of the petroleum products. Given the potential for degradation of resources, there is interest in understanding the structure and source of these communities, and the metabolisms they are employing^2^. Clostridiales are frequently found in oil reservoir surveys^4–7^, including in systems with temperatures greater than 50 °C^1,8,9^. Many observed Clostridiales are spore formers, which may give them an advantage in persisting in these systems with high temperatures, pressures, and salinity. However, this ability also means that they are often overlooked in regards to their influence on the *in-situ* microbial community, as it is unknown if these organisms are active in the reservoir or present in a dormant, sporulated form. Some clostridial species have been implicated as reservoir souring culprits^9^, yet many reservoir descriptions lack any explanation of clostridial activity subsurface^8,10–14^. As such, their role in the reservoir microbial community remains poorly understood. Most Clostridiales employ fermentation as a metabolic strategy, however, many *Desulfotomaculum* species respire using sulfate reduction, and some *Desulfotomaculum* have been shown to directly utilize aromatic hydrocarbons (e.g., toluene, m-xylene, o-xylene) as carbon and energy sources^15^. Hydrocarbon enrichment cultures from oil reservoirs have stimulated clostridial lineages^16^, and clostridial hydrocarbon degradation genes have been observed in surficial oil-contaminated environments^17^. Previous metagenomic studies of oil reservoirs show clostridial lineages containing genes initially classified as hydrocarbon degradation genes, but well-characterized anaerobic hydrocarbon-degrading genes, *bssA* and *assA*, were not detected. Instead, robust signatures of polysaccharide, peptide, and fatty acid degradation were seen as well as robust pathways of sugar fermentation^4^. To our knowledge, few studies have assessed the portion of sporulated and vegetative cells of Clostridiales in oil reservoirs.

In this study, we sampled production wells from Galveston 209, a mature oil field ~32 km south of Galveston Island, Texas. Well temperatures of this oil field range from 80 to 160° Celsius. Discovered in 1983, the field was extensively developed off two platforms (A & B) with a cumulative production over 23 MBO (million barrels of oil) and 300 GCF (gas cubic feet) out of stacked pay of Lower to Middle Miocene shallow marine sandy reservoirs (~2.1 to 4.6 km depth) with outer shelf mudstone seals, all deposited in a wave-dominated deltaic setting. Production data indicates that there is a strong water drive through well-connected, continuous reservoirs. Traps are in low-relief fault-dependent closes on basinward-dipping listric normal faults. In this study, microbial communities in the produced fluids (mixture of oil and water) of these hot wells were examined via metagenomics. Functional annotation and the generation of metagenome assembled genomes (MAGs) were used to examine potential community metabolisms. A novel clostridial lineage which we propose to name ^U^*Petromonas tenebris* was the dominant microbial signature in this subsurface environment, and *in-situ* replication of this organism can be detected via metagenomics.

## Methods

### Collection of produced fluids

Produced fluids were collected using sterile technique at the drilling rig offshore Texas. Fluids came from 4 distinct wells that had temperatures ranging from 88 to 102° C (Table 1). Produced fluids were filtered through a Sterivex filter until the filter clogged, which was seen between 300-400 mL of fluid. The Sterivex filter was frozen immediately and stored at −80 °C until use.

**Table 1:** Selected reservoir produced fluid (water) chemical concentrations and reservoir temperatures.

Chemistry	**Reservoir Well**			
ppm, wt/vol	**A2**	**B6**	**B7**	**B9**
Cl^−^	30034	28298	39183	39106
NO_3_^−^	<1	<1	<1	<1
PO_4_^2−^	<1	<1	<1	<1
SO_4_^2−^	9	22	38	31
Glycolate	7	25	26	19
Formate	3	8	8	6
Acetate	626	1041	806	606
Propionate	43	88	105	87
Butyrate	9	18	14	11
Valerate	3	6	4	4
Bicarbonate	297	294	206	85
Ba	11.4	3.9	3.8	6.7
Ca	512	578	1206	1323
K	151	120	216	199
Mg	101	122	308	361
Na	21116	19840	25653	25298
Si	21.7	18.9	14.8	16.3
Temperature (°C)	102	91	88	91

### DNA extraction and sequencing

DNA was extracted using a modified version of the Qiagen PowerWater Sterivex filter extraction kit^18^. DNA was checked versus a blank control extraction for bacterial PCR products, and once it was determined that DNA was amplifiable and contained a microbial signal, it was sent for metagenomic library preparation and sequencing via Illumina HiSeq at the University of Delaware Genomic Sequencing Facility. Raw sequences and MAGs for this project are deposited at NCBI under Bioproject PRJNA578106.

### Quality trim and assembly

Raw Illumina reads were quality trimmed in CLCBio Workbench version 7.5.1 (Qiagen), with the following parameters: removal of low quality sequence (limit = 0.0016, but rounded to 0.002 by CLCBio, which represents a Phred score of 36 or better); removal of ambiguous nucleotides: No ambiguous nucleotides allowed; removal of terminal nucleotides: 2-12 nucleotides from either end to minimize sequencing errors and enriched 5mers; removal of sequences on length: minimum length 60 nucleotides. Whenever one read of a read pair was excluded due to the quality trim, the entire pair was excluded. Trimmed, paired reads were assembled using IDBA-UD version 1.1.1. with the following settings:–mink 40–maxk 120–step 20–min_contig 300^19^. The resulting scaffolds were then used for further genome binning of each reservoir metagenome.

### Phylogeny

The phylogeny of metagenome community members was determined using EMIRGE (Expectation-Maximization Iterative Reconstruction of Genes from the Environment) based on the reconstructed 16S rRNA gene sequences from unassembled data^20^. A maximum likelihood phylogenetic tree of 16S rRNA gene was inferred from these sequences using Mega version 5 using default parameters for alignment and tree construction with 500 bootstrap replicates^21^.

### Metagenome-assembled genomes (MAGs)

Metagenome assembly of individual samples were subjected to binning using MaxBin version 1.4.2 with the max iteration of 200^22^. The taxonomical uniqueness of each resulting MAG was initially determined using Phylosift version 1.0.1^23^ with the default parameters. The level of potential contamination and strain heterogeneity in each MAG was evaluated using CheckM 1.0.6 with the “lineage_wf” option^24^. The VizBin program^25^ was then used to visually refine the MAGs to minimize outlier scaffolds. Close relative genomes of the clostridial MAGs were downloaded from NCBI (*C. sporogenes* DSM14501 NZ_FRAJ00000000.1 and *P. caminithermalis* DSM15212 NZ_FRAG00000000.1). Average nucleotide identity (ANI) between the MAGs and the two reference genomes were calculated using PyANI^26^ implemented in Anvio v5.5^27^, by following the procedure described here (http://merenlab.org/2016/11/08/pangenomics-v2/). Pair-wise average amino acid identity (AAI) was calculated as one-way AAI and two-way AAI using the online tool AAI calculator (http://enve-omics.ce.gatech.edu/aai/).

A collection of 16 ribosomal proteins^28^ from each MAG were extracted using the Geneious software (Biomatters, Auckland, New Zealand) from the PROKKA annotation. Also included for phylogenetic comparison are genomes from closely related microbial groups downloaded from National Center for Biotechnology Information (NCBI): *E. coli, H. congolese, T. gondwanense, D. alkaliphilum, C. cellulovorans, C. hydrogeniformans, C. acetium, C. forminaceticum, C. sporogenes, P. caminithermalis, M. halophilus*. Ribosomal proteins were concatenated and aligned with MAFFT v7.392^29^ for each of the recovered genomes. Only those genomes that had over 50% of the ribosomal proteins were used in the analysis. The alignment output was used to generate maximum likelihood phylogenetic trees with 100 iterations using FastTree v2.1.11^30^.

### Functional annotation

PROKKA (prokaryotic annotation) version 1.11 was used to annotate the metagenomes and MAGs^31^. COG (Clusters of Orthologous Groups) data was obtained via a local copy of the RAMMCAP (Rapid analysis of Multiple Metagenomes with Clustering and Annotation Pipeline) using the updated version of the 2014 COG database from NCBI^32^.

### Metabolic pathways

The presence or absence of functional genes in metabolic pathways of both cultured metagenome and MAGs were predicted using the BlastKOALA web service provided by the KEGG: Kyoto Encyclopedia of Genes and Genomes website (http://www.kegg.jp/blastkoala/), between July and October, 2017^33^.

### Carbohydrate enzymes

The Carbohydrate Active Enzymes (CAZY) annotation was performed via HMMER searches against the dbCAN release 4.0 HMM (hidden Markov model) database (downloaded from http://cys.bios.niu.edu/dbCAN/), and based on the CAZyDB released on March 17, 2015^34–36^.

### Estimation of growth

Growth signatures were created with the iRep program, which estimates the proportion of actively replicating cells by comparing the read recruitment to the origin of replication^37^.The program was run with default settings.

## Results

Metagenomes were generated using DNA extracted from four samples taken from produced fluids from an oil field located in the Gulf of Mexico, offshore Texas. The reservoirs in this formation are hot and salty, with temperatures ranging between 88-102 °C, salinity values exceeding 28% and sulfate values below seawater levels. Four separate wells access likely connected reservoir material in this system, and each well has somewhat similar but distinct geochemical conditions (Table 1).

We generated metagenomic sequencing data from each well, and the metagenome assembly resulted in 9,600–34,300 contigs varying in total length of 18–43 million basepairs (Table 2).

**Table 2:** Metagenome Statistics.

Metagenome	A2	B6	B7	B9
Total ng of DNA extracted	77	58	172	34
Basepairs DNA Sequenced (Gbp)	9.70	11.84	10.67	9.60
Basepairs after Quality Control (Gbp)	4.37	4.89	4.92	3.90
Basepairs in Assembly	17,973,987	22,158,355	42,651,179	39,671,450
Number of Contigs	9,646	14,826	34,316	25,137
Number of Genes	16,297	20,414	40,930	37,663
Unique 16S rRNA genes (EMIRGE)	6	6	14	13

**Figure 1:**
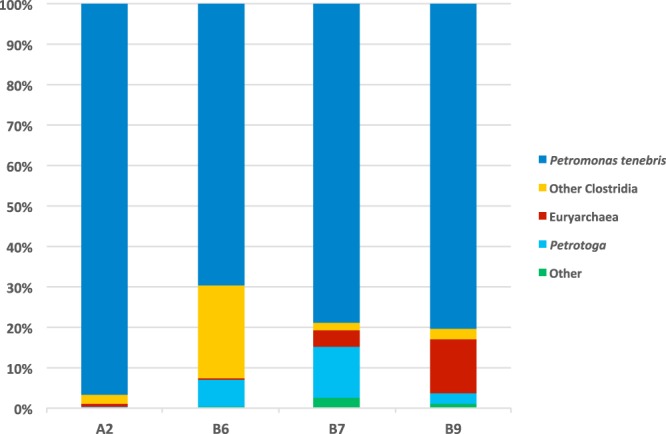
Taxonomic classification of reservoir metagenomes based on 16S rRNA gene sequences reconstructed from EMIRGE analysis of metagenomic data. Other Clostridia include *Desulfallas* species. Euryarchaea includes *Methanothermococcus*. *Petrotoga mobilis* was the only *Petrotoga* species found.

**Figure 2:**
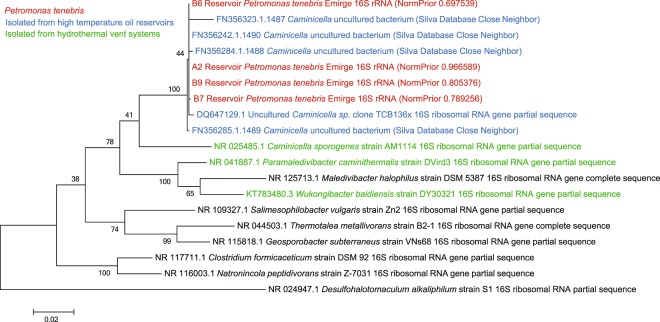
Maximum likelihood tree for ^*U*^*Petromonas tenebris* 16 S rRNA sequences from the reservoirs. The sequences were generated from the EMIRGE program from unassembled Illumina reads (red), or were obtained from the Silva 16 S RNA database or NCBI (black). Relatives from similar environments such as high temperature oil reservoirs (blue) and hydrothermal vent systems (green) are indicated. Bootstrap values were generated from 500 replicates. The scale bar represents substitutions per position.

Based on 16S rRNA gene sequences reconstructed by EMIRGE, the most abundant organism in each metagenome was the unique lineage, ^*U*^*Petromonas tenebris*, which comprised from 69.8% to 96.7% of the microbial community (Fig. 1). *Petrotoga, Geotoga*, Euryarchaea and other members of Clostridiales were less abundant members of the community (Fig. 1). The closest relatives were uncultivated lineages found in other oil reservoirs (Fig. 2).

Phylogenetic analysis suggested that the nearest cultured relatives of the dominant 16 S rRNA gene from the oil reservoirs were *Caminicella sporogenes* and *Paramaledivibacter caminithermalis (*formerly *Clostridium caminthermale)*, both of which are moderately thermophilic and halophilic and were initially isolated from deep-sea hydrothermal vent systems (Fig. 2).

Metagenome assembled genomes (MAGs) of ^*U*^*Petromonas tenebris* lineages were recovered. These high-quality MAGs were 99.2% to 100.0% complete with <5% contamination, as assessed based on single-copy genes by CheckM (Table 3). The estimated sizes of the genomes range between 2.7–3.0 Mb, which ranges in between the relatives of *Ca. sporogenes* (2.5 Mb) and *P. carminithalis* (4.1 Mb). The number of annotated genes of the MAGs ranged from 2,815–2,959 (Table 3).

**Table 3:** ^*U*^*Petromonas tenebris* MAG statistics and comparisons to cultured relative genomes.

**MAG or Genome source**	**A2**	**B6**	**B7**	**B9**	***Ca.sporogenes***	***P.caminthermalis***
Size (bp)	2,946,899	3,033,216	2,689,941	2,893,964	2,473,920	4,060,620
Completeness %	100.0%	100.0%	100.0%	99.2%	—	—
Implied Size (bp)^a^	2,946.899	3.033,216	2,689,941	2,917,302	2,473,920	4,060,620
Contamination %	4.14%	1.65%	1.42%	1.65%	—	—
Strain Heterogeneity	0.00	0.00	0.00	0.00	—	—
Number of Contigs	121	85	146	140	43	155
Number of Genes	2,874	2,959	2,815	2,844	2,422	3,378
**versus** ***C. sporogenes***						
Number of COGs	1,514	1,526	1,514	1,510	1,355	
COGs Unique to MAG	323	330	322	315	—	
COGs Same as Genome	1,191	1,196	1,192	1,195	1,355	
**versus** ***P. caminithermalis***						
Number of COGs	1,514	1,526	1,514	1,510		1,375
COGs Unique to MAG	201	204	193	189		—
COGs Same as Genome	1,313	1,322	1,321	1,321		1,375

^a^Implied genome size equals MAG size divided by completeness.

**Figure 3:**
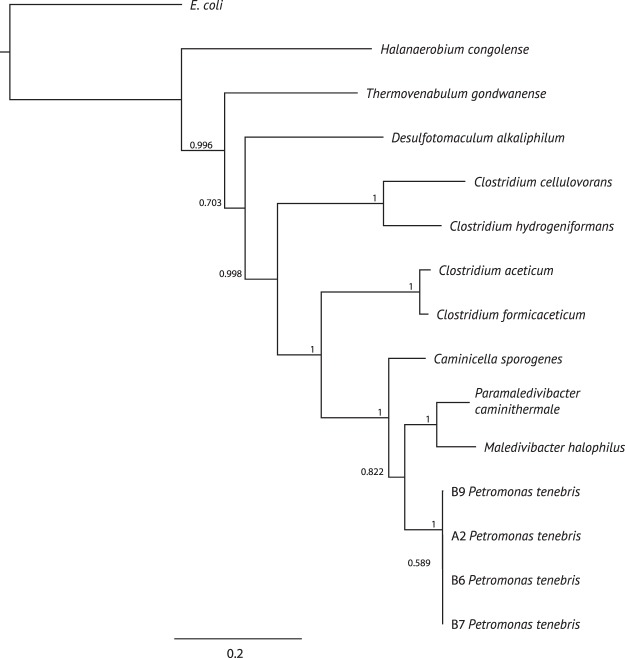
Phylogeny of ^*U*^*Petromonas tenebris* and related genomes based on the concatenated 16 ribosomal proteins: RpL2, 3, 4, 5, 6, 14, 15, 16, 18, 22, and 24, and RpS3, 8, 10, 17, and 19^28^. The tree was reconstructed using the maximum likelihood algorithm with 100 iterations.

Phylogenetic analysis of concatenated ribosomal protein sequences shows that the clostridial MAGs found in these reservoir samples are most closely related to *Caminicella sporogenes* and *Paramaledivibacter caminithermalis*, along with *Maledivibacter halophilus* (Fig. 3). These closely related organisms form a group of thermophilic and halophilic Clostridia within the Clostridiales^38,39^. Both 16S rRNA gene and concatenated ribosomal protein sequences were similar for each of the reservoir MAGs suggesting that the similar organisms were present in all four reservoir samples (Figs. 2 and 3).

Average nucleotide identities (ANI) are 99-100% among the 4 MAGs, but the MAGs share only 74-75% ANI and 51-61% average amino acid identity (AAI) with the cultured genomes (Table 4).

**Table 4:** ANI and AAI values of ^*U*^*Petromonas tenebris* MAGs versus isolate genomes.

		*Ca.sporogenes*	*P.caminithermalis*	A2	B6	B7	B9
Average nucleotide Identity ANI	*Ca. sporogenes*	—	75	75	75	75	75
	*P. caminithermalis*	75	—	74	74	74	74
	A2	74	74	—	100	100	100
	B6	75	74	100	—	99	100
	B7	75	74	100	100	—	100
	B9	75	74	100	100	100	—
Average Amino acid identity AAI 1 way (2 way)	*Ca. sporogenes*	—	51.5	56.6	56.3	57.1	57.1
	*P. caminithermalis*	61.1 (67.3)	—	57.9	57.3	58.5	58
	A2	60.2 (69.4)	51.8 (66.8)	—	95.8	95.4	95.5
	B6	60.8 (69.4)	52.0 (66.7)	97.3 (99.7)	—	96.2	96.7
	B7	60.4(69.5)	51.9 (67.1)	93.6 (99.6)	93.1 (99.6)	—	94.3
	B9	60.8(69.8)	51.9 (67.0)	94.8 (99)	94.5 (99.6)	95.3 (99.7)	—

As such, we provisionally name the organism ^*U*^*Petromonas tenebris* as the MAGs fit the suggested metrics for establishment of a new genus and species^40^. The name comes from “petra” (rock), “monas” (single celled organism) and “tenebris” (dark) since the phylogenetic trees show relatives of these organisms are all found in oil reservoirs, the ^U^ indicates its uncultivated status.

**Figure 4:**
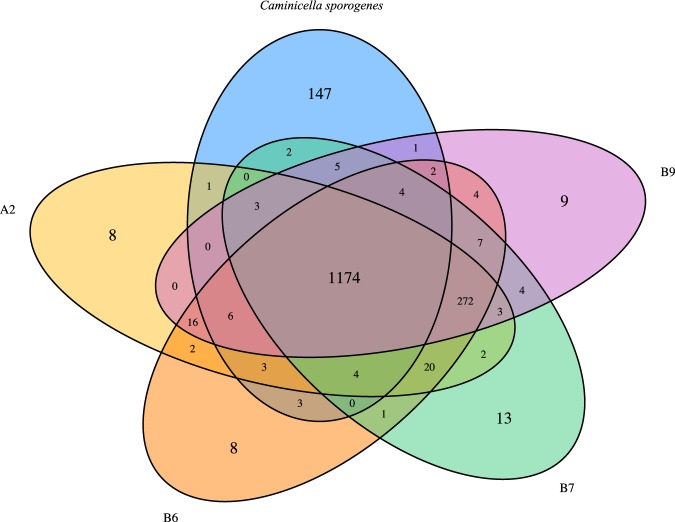
Clusters of Orthologous Groups shared among the ^*U*^*Petromonas tenebris* MAGs B9, A2, B6, B7 and the *Ca. sporogenes* genome. Unique genes are seen in each MAG: 9 in B9, 8 in A2, 8 in B6 and 13 in B7. There are 147 genes unique in *Ca. sporogenes*.

The analysis of Clusters of Orthologous Groups (COGs) shared with either *Ca. sporogenes* and *P. carminithermalis* show that individual MAGs of ^*U*^*Petromonas tenebris* have different levels of overlap with cultured relatives, with an average of 323 and 197 unique genes, respectively (Table 3). Genes present in the MAGs that are different than those found in the isolate genomes include a number of oxidoreductases, including Fe-S oxidoreductase, citrate lyase, and CO dehydrogenase-CoA synthase subunits. There were also between 8-13 COGs unique to each MAG that did not appear in any other MAG or cultured genome (Fig. 4). These unique genes were primarily housekeeping genes except that ^*U*^*P. tenebris* B7 had a unique COG1719, which is a predicted hydrocarbon binding protein.

**Figure 5:**
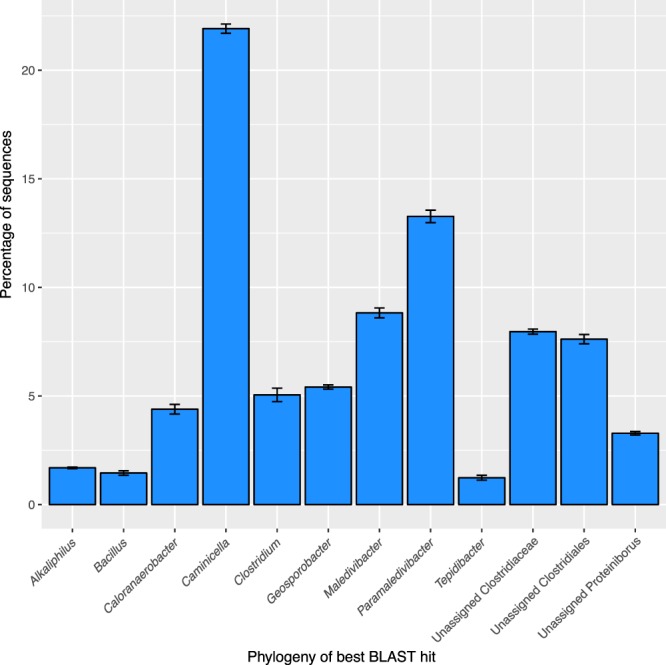
Potential source phylogenies for genes present in the ^*U*^*Petromonas tenebris* bins as determined by DarkHorse, which retrieves the phylogeny of the best BLAST hit for each sequence. Error bars represent standard deviations among the four bins. Nearly 25% of the genes represent the *Caminicella* genus, the remainder appear more closely related to other groups within the Firmicutes.

The potential phylogenetic history of functional genes in the reservoir MAGs was examined to determine if they had any unusual evolutionary histories. Each gene was individually examined via BLAST and the taxonomy of the best hit was recorded. About 48% of the coding genes in the reservoir MAGs appear to have originated within the *Caminicella*, *Maledivibacter*, and *Paramaledivibacter* genera, with the remainder coming from other Clostridiales or Firmicutes groups (Fig. 5).

Many metabolic pathways are shared between the reservoir MAGs and their cultured relatives. Shared core metabolisms include butyrate fermentation, sporulation, and common two component regulatory systems involving temperature, salt stress, chemotaxis, and flagella regulation. However, many differences between the MAGs and the closely related cultured organisms exist. For example, there are differences in Carbohydrate Active Enzymes (CAZY) between each of the cultured genomes and between the cultured genomes and the reservoir MAGs (Table 5). The reservoir MAGs have unique genes and transporters involved in the metabolism of sucrose which are not present in the cultured genomes. Conversely, the cultured isolate genomes are capable of processing extracellular cellobiose and xylan/xylose, while the reservoir MAGs lack these genes.

**Table 5:** Differences in Carbohydrate Active Enzymes (CAZY) between the ^*U*^*Petromonas tenebris* MAGs and the *Caminicella sporogenes* and *Paramaledivibacter caminithermalis* genomes.

**Family**^a^	**Description**	**Carbohydrate Family present in**		
		*C. sporogenes*	*P. caminithermalis*	*P. tenebris* MAGs
GH2	β-galactosidase, β-mannosidase, β-glucuronidase, others		X	
GH3	β-glucosidase, xylan 1,4-β-xylosidase, and others	X	X	
GH4	α-glucosidase, α-galactosidase, and others	X	X	
GH9	Endoglucanase, cellobiohydrolase, β-glucosidase, others		X	
GH32	Sucrose invertases, also hydrolysis of fructose			X
GH33	Sialidases	X		
GH42	β-galactosidases, α-L-arabinosidase, and others			X
GH46	Chitosanase		X	
GH63	Mannosyl oligosaccharide glucosidase, α-glucosidases		X	
GH78	Hydrolysis of alpha-L-rhamnosides	X		
GH84	N-acetyl β-glucosaminidase, hyaluronidase		X	
GH94	Phosphorylases that cleave β-glycosidic bonds			X
GH100	Alkaline and neutral invertases	X		
GH113	β-mannanase		X	
GH120	β-xylosidase		X	
CBM34	Granular starch binding function	X		
CBM37	Broad binding specificity, including xylan, chitin, others	X	X	
CBM39	Beta-1,3-glucan binding function			X
CBM45	Alpha glucan binding function, including starches			X
CBM49	Binding to crystalline cellulose	X	X	
CE1	Acetyl xylan esterase, cinnamoyl esterase, others		X	
CE7	Acetyl xylan esterase, cephalosporin-C deacetylase		X	
CE14	Glucopyranoside deacetylase, diacetylchitobiose deacetylase, etc.		X	
GT14	Number of beta-glycotransferases	X	X	
GT27	Polypeptide α-N-acetylgalactosaminyltransferase	X		
GT32	Alpha-1,6-mannosyltransferase, and others			X
GT84	Cyclic beta-1,2-glucan synthase			X
GT94	Lipid-A-disaccharide synthase	X		

^a^GH = Glycoside Hydrolases, CBM = Carbohydrate Binding Modules, CE = Carbohydrate Esterases, and GT = Glycosyltransferases.

Other key metabolic differences include a complete Wood–Ljungdahl CO_2_ fixation pathway found in three of the four reservoir MAGs, but not in the other genomes (Table 6). COGs of the key enzyme, CO dehydrogenase/acetyl CoA synthase, noted above, were not found in the cultured genomes. Certain sulfur metabolism genes are present in the reservoir MAGs but not in the cultured genomes. These include anaerobic sulfite reductase (ASR), adenosine 5′-phosphosulfate reductase (APR), and sulfite reductase (ferredoxin). ASR is typically part of the assimilatory sulfate reduction pathway, and APR is typically present in dissimilatory pathways, but neither of these pathways is complete in the MAGs. In addition, neither of the *qmoABC* and *dsrMKJOP* electron transport complex genes typically found in sulfate reducers are present in the MAGs. As a result, it does not appear that any of the reservoir MAGs and cultured genomes can perform dissimilatory sulfate reduction for energy conservation, but instead use sulfur compounds as electron sinks for fermentation.

**Table 6:** Differences in metabolism between the MAGs and cultured isolate genomes.

**Metabolism**	***Caminicella sporogenes***	***Paramaledivibacter*** ***caminithermalis***	**A2** ***P. tenebris*** **MAG**	**B6** ***P. tenebris*** **MAG**	**B7** ***P. tenebris*** **MAG**	**B9** ***P. tenebris*** **MAG**
**Wood-Ljundahl Pathway**						
Complete pathway			X	X	X	
**Sulfur metabolism**						
Anaerobic Sulfite Reductase			X	X	X	X
Aden-5’phosphosulfate Reduct			X	X	X	X
Sulfite reductase(ferrodoxin)			X	X	X	X
**Butanoate Metabolism**						
Pyruvate <–> Acetyl CoA	X	X	X	X	X	X
Acetyl CoA <–> AcetoacetylCoA	X	X	X	X	X	X
Acetyl CoA < –> Butanoate	X	X	X	X	X	X
Acetyl CoA <–> Butanol	X	X	X	X	X	X
**Starch and Sucrose**						
Extracellular Cellubiose	X	X				
Starch/Glycogen–> Glucose		X	X	X	X	X
Sucrose–> Glucose			X	X	X	X
**Membrane Transport**						
Phosphonate			X	X	X	X
Liposaccharide			X	X		X
Glycine betaine/proline			X	X	X	X
Cobalt			X	X	X	X
Nickel			X	X	X	X
Galactitol			X	X	X	X
Mannitol			X	X	X	X
Sorbital			X	X	X	X
Sucrose			X	X	X	X
Amino Acid	X	X				
Branched Chain Amino Acids		X				
Bacitracin/Lantibiotics		X				
Cellubiose/diacetylchitobiose	X	X				
Complete D-xylose		X				
Galactose oligomer	X					
N-acetyl-D-glucosamine	X	X				
**Two-component systems**						
Temperature	X	X	X	X	X	X
Salt Stress	X	X	X	X	X	X
Chemotaxis	X	X	X	X	X	X
Flagella regulon genes	X	X	X	X	X	X
Tricarboxylate transport	X	X				
Citrate formation genes		X	X	X	X	X

Both *Caminicella sporogenes* and *Paramaledivibacter caminithermalis* contain a glycyl radical enzyme with the same active site as the gene annotated as a pyruvate formate lyase (*pflD*, locus tag AF1449) found in *Archaeoglobus* species, which may be used to anaerobically metabolize some hydrocarbons^41^. However, the annotated pyruvate formate lyase genes found in the ^*U*^*Petromonas tenebris* MAGs contain a different active site that was found in more typical carbohydrate fermenters, leading to the conclusion that these are indeed typical pyruvate formate lyase genes used in the majority of anaerobic bacteria^41^. We examined the metabolic profile of the MAGs via standard annotation by PROKKA and also via individual BLAST analysis for known anaerobic hydrocarbon degradation genes^42,43^. We found no evidence for other anaerobic hydrocarbon metabolism pathways, except that the gamma subunit of acetophenone carboxylase, an enzyme in the ethylbenzene degradation pathway, was present in each of the MAGs. However, the gene encoding ethylbenzene dehydrogenase, the initial enzyme in this pathway^44^, could not be found. As such, based on current genomes and annotations, ^*U*^*Petromonas tenebris* seems not to be capable of utilizing hydrocarbons. We do caveat that as seen in the *Archaeoglobus* case^41^, genes may exist for hydrocarbon degradation that are either unannotated or misannotated and full proof cannot be given until a culture is tested.

To determine if the ^*U*^*Petromonas tenebris* MAGs came from spores or active, vegetative cells we estimated the index of replication (iRep) of the MAGs in the four oil reservoirs. The resulting iRep indexes ranged from 1.35-1.40 for the reservoir MAGs. For reference, this iRep value is comparable to the median values seen in other environments, including soil (1.34) and human gut systems (1.37–1.42)^37^, and should be interpreted as a measurement indicative of growth. The value indicates 35–40% of these cells were replicating *in situ* at time of sampling.

## Discussion

The hot and salty oil reservoirs described here represent a challenging environment for microbial growth. Using metagenomic analysis, we found the dominant species in these reservoirs is related to the thermophilic *Caminicella*/*Paramaledivibacter* clades of thermophilic and halophilic Clostridiales, and form a distinct clade with other uncultured organisms found in high-temperature oil reservoirs (Fig. 2). Due to these unique features, we propose to name this lineage ^*U*^*Petromonas tenebris* (Figs. 2 and 3). Related species have been detected in other oil well systems, including *Ca. sporogenes* in Oman at temperatures near 60 °C^45^ and formation water from the high temperature Ekofisk oil field in the North Sea^46^. While these relatives do not grow at temperatures as high as observed in this environment, hyperthermophilic clostridial species have been documented from oil wells previously^47^. However, no genomic information is available for comparison to these MAGs.

It is unlikely that these MAGs represent infrastructure contaminants or sporulated, inactive cells. First, 35–40% of these cells were in the process of replication. Additionally, the DNA was readily extractable and this species dominated the community, also suggesting it was not heavily sporulated. Considering the apparent lack of hydrocarbon consumption by these cells, it is plausible that clostridial spores present in the subsurface may have germinated as the oil seeped upward from hotter source rocks to a slightly cooler reservoir formation, where water was present. However, we interpret the low diversity of the community to be reflective of the challenging *in-situ* environment. We cannot refute the hypothesis that spores may be germinating en route to oil processing inside pipelines, however, we note that communities which show infrastructure influence are typically much more complex, reflecting the increase in electron acceptors and metals available within pipelines, as well as industrially introduced materials^4^. As such, the entirety of the data presented suggests these are active *in-situ*.

Other organisms in this system in lower abundance (Fig. 1) include *Methanothermococcus* methanogens which use hydrogen and formate as electron donors^48^, and *Desulfallas* species such as *D. gibsonae* and *D. geothermicum*, which utilize simple organic compounds, including some carbohydrates and/or fatty acids, and alcohols such as ethanol, propanol, and butanol, as electron donors and sulfur compounds as electron acceptors, producing carbon dioxide or acetate as end products^49^. Also present is *Petrotoga mobilis*, which is a fermenter of a variety of carbohydrates including xylan^50^. This system may be a syntrophic methanogenic system in which the ^*U*^*Petromonas* and *Petrotoga* ferment complex organic compounds, with *Desulfallas* and *Methanothermococcus* scavenging the fermentation products. Based on the analysis of genomes, no alkane metabolizing partner has been detected, compared to other syntrophic methanogenesis systems that were explored via enrichment cultures^5,51^. Therefore, ^*U*^*Petromonas tenebris* represent one of the keystone members of the microbial community inhabiting this harsh environment.

Despite being retrieved from a similar geologic formation, the ^*U*^*Petromonas tenebris* MAGs show some distinctions between wells. In particular, they contained between 8-13 unique genes per genome (Fig. 4). They were clearly differentiated from their nearest cultivated neighbors by both ribosomal sequences (Figs. 2 and 3) and genome size and content (Tables 1 and 3, Fig. 4). The variations in genome all seem to be of clostridial origin (Fig. 5). Overall the MAGs in this reservoir system represent a single species, supported by phylogenies (Figs. 2 and 3) and ANI calculation (Table 2), despite the unique genome values mentioned previously.

The ^*U*^*Petromonas tenebris* MAGs are the most abundant organisms in this reservoir system, they show signatures of replication, and potential end products of their fermentative metabolism have built up in this system. Yet they lack potential hydrocarbon processing pyruvate formate lyase genes seen in the cultured relatives, and none of the other less abundant organisms have signatures of directly processing hydrocarbons either. Nevertheless, the ^*U*^*Petromonas tenebris* in this reservoir have become a dominant species in the environment, likely through fermentation, are growing *in-situ* and may be responsible for the increase of organic acids in the reservoirs. Due to their potential influence in reservoirs, Firmicutes and clostridial relatives should be regarded as key players in reservoir microbiomes, not only as sporulated, inactive cells. Furthermore, presence of these organisms in produced fluids, drilling fluids, or naturally seeped fluids could be an indicator of the temperature of a connected subsurface reservoir, with applications to oil and gas exploration and development^52^.
